# The Influence of Gene-Gene and Gene-Environment Interactions on the Risk of Asbestosis

**DOI:** 10.1155/2013/405743

**Published:** 2013-07-25

**Authors:** A. Franko, V. Dolžan, N. Arnerić, M. Dodič-Fikfak

**Affiliations:** ^1^Clinical Institute of Occupational Medicine, University Medical Centre, Ljubljana, Poljanski nasip 58, 1000 Ljubljana, Slovenia; ^2^Pharmacogenetics Laboratory, Institute of Biochemistry, Faculty of Medicine, University of Ljubljana, 1000 Ljubljana, Slovenia

## Abstract

This study investigated the influence of gene-gene and gene-environment interactions on the risk of developing asbestosis. The study comprised 262 cases with asbestosis and 265 controls with no asbestos-related disease previously studied for *MnSOD*, *ECSOD*, *CAT*, *GSTT1*, *GSTM1*, *GSTP1*, and *iNOS* polymorphisms. Data on cumulative asbestos and smoking were available for all subjects. To assess gene-gene and gene-environmental interactions, logistic regression was used. The associations between *MnSOD* Ala −9Val polymorphism and the risk of asbestosis and between *iNOS* genotypes and asbestosis were modified by *CAT* –262 C > T polymorphism (*P* = 0.038; *P* = 0.031). A strong interaction was found between *GSTM1*-null polymorphism and smoking (*P* = 0.007), *iNOS* (CCTTT)*_n_* polymorphism and smoking (*P* = 0.054), and between *iNOS* (CCTTT)*_n_* polymorphism and cumulative asbestos exposure (*P* = 0.037). The findings of this study suggest that the interactions between different genotypes, genotypes and smoking, and between genotypes and asbestos exposure have an important influence on the development of asbestosis and should be seriously considered in future research on occupational/environmental asbestos-related diseases.

## 1. Introduction

The findings of the studies indicate that, in addition to asbestos exposure, the genetic factors may influence the development of asbestosis [1–8]. 

The reactive oxygen and nitric species (ROS and RNS) such as superoxide anion (O_2_^∙−^), hydrogen peroxide (H_2_O_2_), hydroxyl radical (OH^*∙*^), and nitric oxide (NO) are suggested to be involved in the pathogenesis of this disease [9–11]. Several specific enzyme systems contribute to the disposition of ROS and RNS. Superoxide dismutases (SODs) like manganese SOD (MnSOD) and extracellular SOD (ECSOD) and catalase (CAT) together with glutathione peroxidases represent an important line of the primary enzyme defence system against ROS. Superoxide dismutases catalyse the dismutation of O_2_^∙−^ to H_2_O_2_ and oxygen (O_2_), whereas CAT subsequently catalyses the conversion of H_2_O_2_ to water (H_2_O) and O_2_ [10, 12, 13]. Other important enzymes involved in the detoxification of ROS and RNS are glutathione S-transferases (GSTs) such as GSTM1, GSTT1, and GSTP1 which catalyse the conjugation of reduced glutathione to different electrophiles [14–16]. The asbestos fibres have also been shown to upregulate the activity of inducible nitric oxide synthase (iNOS) and thus the production of NO by alveolar macrophages and pulmonary epithelial cells, which may play an important role in the initiation and progression of asbestosis [11, 17, 18].

The genes coding for all these enzymes are polymorphic [1–3, 12, 16, 19–21]. The most common single-nucleotide polymorphism (SNP) of the *MnSOD *gene results in alanine (Ala) to valine (Val) substitution (Ala-9Val); of the *ECSOD *gene results in arginine (Arg) to glycine (Gly) change (Arg213Gly); and of the *CAT* gene results in cytosine (C) to thymine (T) substitution (−262C > T) [12, 13, 22]. The *GSTM1 *and *GSTT1* genes exhibit null polymorphism due to gene deletion [3, 16]. In the *GSTP1* gene, two functional SNPs cause isoleucine (Ile) to Val substitution (Ile105Val) and Ala to Val change (Ala114Val) [16, 20]. Based on the presence of polymorphisms in both codons (105 and 114), *GSTP1* genotypes may be combined into groups with a presumed high, intermediate, or low conjugation capacity of the enzyme, as described previously [5, 20]. Regarding *iNOS*, one of the most frequently investigated polymorphisms is the CCTTT pentanucleotide repeat ((CCTTT)*_n_*) in the promotor region [21, 23].

The associations between asbestosis and different genetic polymorphisms have been investigated in several studies [2–8, 14]. However, to our knowledge and the available literature, the interactions between genotypes and environmental factors and between different genotypes have not been studied so far in association with asbestosis. This paper presents the influence of interactions between different genotypes (*MnSOD* Ala −9Val, *ECSOD* Arg213Gly, *CAT *−262C > T, *GSTT1*-null, *GSTM1*-null, *GSTP1* Ile105Val, and Ala114Val and *iNOS *(CCTTT)*_n_*), between genotypes and smoking, and between genotypes and cumulative asbestos exposure on the risk of developing asbestosis.

## 2. Methods

The participants in the nested case-control study were selected from a cohort of 2,080 workers occupationally exposed to asbestos who were presented at the State Board for the Recognition of Occupational Asbestos Diseases at the Clinical Institute of Occupational Medicine in Ljubljana in the period from January 1, 1998 to December 31, 2003. In this cohort, a total of 356 subjects were diagnosed with asbestosis. All these subjects were included in the present study and represented the cases. A group of 356 controls matched by gender and age with no asbestos-related disease was selected from the same cohort of workers occupationally exposed to asbestos. However, among the selected cases, 40 (11.2%) died in the period from the recognition of the occupational disease to the time of the beginning of the study, 2 (0.6%) developed a malignant disease and 52 (14.6%) refused to participate, so the final number of cases included in the study was 262 (73.6%). Among the controls, 29 (8.1%) died, 9 (2.5%) developed a cancer, and 53 (14.9%) rejected taking part in the study, so the final number of controls was 265 (74.5%). 

The information on smoking history was collected for all subjects during an interview using a standardized questionnaire [24, 25]. The data on the cumulative asbestos exposure, expressed in fibres/cm^3^-years, were available for all the subjects from the previous study [25] as all the subjects were occupationally exposed to asbestos in the Salonit Anhovo cement manufacturing plant, Slovenia. To determine the cumulative asbestos exposure, the exposure measurements were available for all jobs. Three different methods of measurement were used: konimeter measuring particles/cm^3^, a gravimetric method measuring milligrams/m^3^, and membrane filter measuring fibers/cm^3^. The exposure estimation included the following main steps: (1) for all production workers, work histories were obtained from the company personnel files; (2) all air measurements, information about product (asbestos or asbestos-cement) for a particular task, the process type (wet or dry), percent of time per task, units used for the air sampling measurements (particles/cm^3^, milligrams/m^3^, and fibres/cm^3^), and department were entered into an ACCESS table for each production job for every year in the study period; (3) operation-specific conversion factors from particle/cm^3^ to fibres/cm^3^ and mg/m^3^ to fibres/cm^3^ were calculated; (4) applying the appropriate conversion factor to the measured and estimated exposure intensities, exposure intensities by job and year were calculated for asbestos for all production workers; (5) the exposure intensity table for production jobs (The Job Exposure Matrix) and the work histories for each production worker were combined using the SAS program to obtain the cumulative exposure for each worker [25]. 

The diagnosis of asbestosis or “no asbestos-related disease” was verified by two groups of experts of the State Board for Recognition of Occupational Asbestos Diseases, following the Helsinki Criteria for Diagnosis and Attribution of Asbestos Diseases [26] and the American Thoracic Society recommendations [27]. According to these recommendations [27], high-resolution computer tomography (HRCT) was used for the radiological diagnosis of the disease. Each group of experts consisted of an occupational physician, a radiologist, and a pulmonologist skilled in the diagnosis of asbestos-related diseases.

PCR-based methods were used for *MnSOD* Ala −9Val, *ECSOD* Arg213Gly, *CAT *−262C > T, *GSTT1*-null, *GSTM1*-null, *GSTP1* Ile105Val and Ala114Val, and *iNOS *(CCTTT)*_n_* genotyping as previously described [4–8].

The statistical analysis followed the standard procedure calculating first the descriptive statistics, *t*-test, *χ* ^2^ test, and univariate logistic regression analysis. Next, multivariate logistic regression modelling, including genotypes, cumulative asbestos exposure, possible confounders, or effect modifiers, was employed. To test the effect modification (interactions), simple categorical models based on stratification were constructed first, followed by logistic regression models using dummy variables. 

## 3. Results

The baseline characteristics (age, gender, smoking status, and cumulative asbestos exposure) of cases and controls are presented in Table 1.

The frequencies of *MnSOD*, *ECSOD*, *CAT*, *GSTT1*, *GSTM1*, *GSTP1*, and *iNOS* genotypes in this cohort were described previously [4–8]. In the control group, all investigated biallelic polymorphisms were in the Hardy-Weinberg equilibrium (*P* > 0.05; data not shown). 

Logistic regression analysis revealed no association between asbestosis and smoking (ever/never) (OR = 0.98, 95% CI = 0.69–1.39), while a significant association was observed between asbestosis and log-transformed cumulative asbestos exposure (OR = 3.21, 95% CI = 2.43–4.23). The results of univariate logistic regression analysis for *MnSOD*, *ECSOD*, *CAT*, *GSTT1*, *GSTM1*, *GSTP1*, and *iNOS* genotypes (unadjusted and adjusted by gender, age, smoking, and cumulative asbestos exposure) were reported in detail in previous studies [4–8] and are summarized in Table 2. 

In a subsequent statistical analysis, no significant change in the risk of asbestosis was observed in numerous multivariate models involving different genotypes, cumulative asbestos exposure and possible confounders, or effect modifiers compared to univariate models (data not shown).

Analysing the interactions between different genotypes, the association between *MnSOD* Ala –9Val polymorphism and the risk of asbestosis was modified strongly by *CAT *–262 C > T polymorphism (Tables 3 and 4). An increased risk of asbestosis was found for the combined *MnSOD* –9Ala/Val and Val/Val genotypes compared to the Ala/Ala genotype only among carriers of *CAT *−262 TT genotype (OR = 2.67, *P* = 0.004) (Table 3). Similarly, the association between *iNOS *(CCTTT)*_n_* polymorphism and asbestosis was modified by *CAT *–262 C > T polymorphism (Tables 3 and 4), where a higher risk of asbestosis for the *iNOS* LL genotype versus the combined SL and SS genotypes was also observed only among those who had *CAT* −262 TT genotype (OR = 5.14, *P* = 0.000) (Table 3). No interaction was found between other investigated genotypes.

Testing the interactions between different genotypes and smoking, *GSTM1*-null polymorphism was shown to modify the association between smoking and asbestosis (Tables 3 and 4), where an increased risk of asbestosis was found only among ever-smokers who had *GSTM1*-null genotype (OR = 1.48, *P* = 0.009) (Table 3). Similarly, the association between smoking and asbestosis was modified by *iNOS *(CCTTT)*_n_* polymorphism (Tables 3 and 4). In this case, an elevated risk of asbestosis was detected for ever smokers with *iNOS *LL genotype (OR = 1.39, *P* = 0.050) (Table 3). Other investigated genotypes showed no interaction with smoking. 

To assess the interactions between the genotypes and cumulative asbestos exposure, simple categorical models that included cumulative asbestos exposure categorized as ≤11.23 fibres/cm^3^-years and >11.23 fibres/cm^3^-years (11.23 fibres/cm^3^-years is the mean cumulative asbestos exposure for the controls) were constructed first. The analysis showed that the association between dichotomized cumulative asbestos exposure and the risk of asbestosis was modified by *iNOS *(CCTTT)*_n_* polymorphism (Table 3). The *CAT* −262 TT and combined *CAT* CT and CC genotypes showed a very different magnitude of association between the cumulative asbestos exposure and risk of asbestosis. In both cases, there was a strong risk of asbestosis, but the risk was still much higher for subjects with *CAT* −262 TT genotype. Next, logarithmically transformed cumulative asbestos exposure as a continuous variable was included in the logistic regression models. In these models, an important interaction was found only for the *iNOS *(CCTTT)*_n_* polymorphism (Table 4), while no modifying effect was observed for other genotypes.

In all presented models, the likelihood ratio test showed that the interaction model is better if compared to the models including only the main effects (*P* < 0.05).

We also included possible confounders (age, gender, smoking, and cumulative asbestos exposure) in models testing the genotype-genotype and genotype-environmental interactions, but there was no important difference in asbestosis risk compared to the models presented (data not shown).

## 4. Discussion 

The already published findings of our study show that *MnSOD *–9Ala/Ala and *GSTP1*105Ile/Ile genotypes significantly increase the risk of developing asbestosis, while a protective effect was observed for *GSTT1*-null genotype [4, 5, 7]. An elevated risk of asbestosis was also observed for the *ECSOD *213Arg/Gly genotype, *CAT* −262 TT genotype and *iNOS *LL genotype, but the results were not significant or borderline significant [6–8]. In this paper, we additionally present the interactions between different genotypes, genotypes and smoking, and between genotypes and cumulative asbestos exposure.

A crucial finding of the current study shows that *CAT *–262 C > T polymorphism strongly modifies the association between *MnSOD* Ala –9Val polymorphism and the risk of asbestosis. As both MnSOD and CAT constitute part of the primary defence system against ROS and catalyse the sequence of reactions in the detoxification of ROS [10, 12, 13], this interaction could be considered as biologically plausible. Similarly, the *CAT *–262 C > T polymorphism has been shown to also modify the association between *iNOS *(CCTTT)*_n_* polymorphism and asbestosis. Considering that ROS and NO have been proposed to cooperate in causing the cytotoxic and mutagenic effects of asbestos fibres [10, 11] and based on the assumptions that NO produced by the catalytic activity of iNOS can function as a protective agent against toxic effects of H_2_O_2_ [28], which is detoxified by CAT [10, 12, 13], and vice versa, that H_2_O_2_ decreases the cytotoxicity of NO [29], this interaction is also a logical and important finding.

According to present knowledge, asbestosis has not been associated with smoking with certainty [30, 31]. Nevertheless, this study demonstrates a strong interaction between *GSTM1*-null polymorphism and smoking, despite the fact that there was no independent association between either *GSTM1*-null polymorphism or smoking and asbestosis risk. The explanation could be that both asbestos and smoking increase the production of ROS [9, 32, 33], which are known to be involved in the pathogenesis of asbestosis [9, 10, 34, 35]. It has been suggested that cigarette smoke and asbestos increase DNA damage and ROS production in pulmonary cells synergistically [32, 33]. Studies have shown that fresh grinding of asbestos fibres and cigarette smoke increase the production of OH^*∙*^ by 2-3 times [33]. In line with these reports and considering the role of *GSTM1* in the defence against ROS [14, 15, 36, 37], this result could also be physiologically explained. An interaction was also observed between smoking and *iNOS *(CCTTT)*_n_* polymorphism. This interaction may be explained by the observation that cigarette smoke is the largest source of NO and can also increase the expression and activity of iNOS [38, 39] and by the suggestion that asbestos fibres may upregulate the activity of iNOS and thus the production of NO, which is believed to be important in the initiation and progression of asbestosis [11, 17].

This study also suggested a modifying effect of *iNOS *(CCTTT)*_n_* polymorphism on the association between asbestosis and cumulative asbestos exposure. This has been proved in the simple categorical model and in logistic regression analysis with logarithmically transformed cumulative asbestos exposure as a continuous variable. Additional studies including more subjects are needed to elucidate whether other genetic polymorphisms modify or confound cumulative asbestos exposure—asbestosis associations. 

In the present study, no bias was introduced by genetic heterogeneity because all the subjects were recruited in a small geographical area with an ethnically homogeneous population [40]. 

In conclusion, the findings of this study suggest that the interactions between different genotypes, genotypes and smoking, and between genotypes and asbestos exposure have an important influence on the development of asbestosis and should be seriously considered in future research on occupational/environmental asbestos-related diseases. 

## Figures and Tables

**Table 1:** Baseline characteristics of cases and controls.

	Cases (*n* = 262)	Controls (*n* = 265)	Test	*P* value
Age in years (mean ± SD)	61 ± 9.40	57 ± SD 9.34	*t* = 5.18	0.000
Gender				
Male [*n* (%)]	186 (71)	183 (69)		
Female [*n* (%)]	76 (29)	82 (31)	*χ* ^2^ = 0.24	0.628
Smoking				
Ever/never smokers [*n*]	117/145	120/145	*χ* ^2^ = 0.01	0.919
Years [mean ± SD]	25.92 ± 13.37	22.90 ± 12.90	*t* = 1.77	0.078
Pack years [mean ± SD]	21.92 ± 15.95	20.99 ± 16.37	*t* = 0.44	0.659
Cumulative asbestos exposure in fibres/cm^3^-years [mean ± SD]	37.67 ± 86.43	11.23 ± 23.47	*t* = 4.78	0.000

**Table 2:** The risk of asbestosis for *MnSOD*, *ECSOD*, *CAT*, *GSTT1*, *GSTM1*, *GSTP1*, and *iNOS* genotypes*.

Genotype	OR (95% CI)				
	Unadjusted	Adjusted by			
		Gender	Age	Smoking (ever/never)	Cumulative exposure
*MnSOD* −9Ala/Ala versus Ala/Val + Val/Val	1.50 (1.01–2.24)	1.49 (1.00–2.23)	1.46 (0.97–2.19)	1.49 (1.00–2.23)	1.48 (0.96–2.28)
*ECSOD* Arg/Gly versus Arg/Arg	1.63 (0.62–4.27)	1.61 (0.61–4.22)	1.49 (0.56–3.96)	1.65 (0.63–4.32)	2.07 (0.72–5.94)
*CAT *−262 TT versus CT + CC	1.36 (0.70–2.62)	1.34 (0.70–2.60)	1.31 (0.67–2.57)	1.37 (0.71–2.66)	1.91 (0.93–3.91)
*GSTM1*-null versus present	1.01 (0.71–1.43)	1.00 (0.70–1.42)	0.94 (0.66–1.35)	0.99 (0.70–1.41)	0.97 (0.67–1.42)
*GSTT1*-null versus present	0.61 (0.40–0.94)	0.62 (0.40–0.94)	0.63 (0.40–0.97)	0.63 (0.41–0.97)	0.60 (0.38–0.96)
*GSTP1* 105Ile/Ile versus Ile/Val + Val/Val	1.52 (1.08–2.15)	1.53 (0.60–1.28)	1.49 (1.04–2.11)	1.54 (1.08–2.18)	1.41 (0.97–2.05)
*GSTP1* 114Ala/Ala versus Ala/Val + Val/Val	0.97 (0.64–1.48)	0.97 (0.64–1.48)	0.99 (0.65–1.53)	0.94 (0.62–1.44)	0.86 (0.55–1.36)
*GSTP1* high versus intermediate + low conjugation capacity	1.49 (1.06–2.10)	1.50 (1.06–2.11)	1.45 (1.03–1.07)	1.50 (1.06–2.13)	1.36 (0.94–1.98)
*iNOS* LL versus SL + SS	1.20 (0.85–1.69)	1.20 (0.85–1.70)	1.19 (0.84–1.69)	1.17 (0.83–1.66)	1.19 (0.82–1.73)

*The table summarizes the results from our previous studies.

**Table 3:** Stratification of *MnSOD* by *CAT*,  *iNOS* by *CAT*, smoking by *GSTM1*, smoking by *iNOS*, and cumulative asbestos exposure (>11.23 versus ≤11.23) by *iNOS*.

	OR	95% CI	*P* value
Stratification of *MnSOD* by *CAT *			
Crude	0.67	0.44–1.01	0.047
* CAT* −262 TT	2.67	0.57–13.07	0.004
*MnSOD* −9Ala/Val + Val/Val versus Ala/Ala			
* CAT* −262 CT + CC	0.59	0.38–0.93	
* MnSOD* −9Ala/Val + Val/Val versus Ala/Ala			
Stratification of *iNOS* by *CAT *			
Crude	1.20	0.85–1.69	0.312
* CAT* −262 TT	5.14	1.30–20.36	0.000
* iNOS* LL versus SL + SS			
* CAT* −262 CT + CC	1.08	0.75–1.55	
* iNOS* LL versus SL + SS			
Stratification of smoking by *GSTM1 *			
Crude	0.98	0.69–1.39	0.357
* GSTM1*-null	1.48	0.92–2.39	0.009
Smoking: ever versus never			
* GSTM1*-present	0.55	0.31–1.00	
Smoking: ever versus never			
Stratification of smoking by *iNOS *			
Crude	0.98	0.69–1.39	0.357
* iNOS* LL	1.39	0.84–2.30	0.050
Smoking: ever versus never			
* iNOS* SL + SS	0.70	0.43–1.31	
Smoking: ever versus never			
Stratification of cumulative asbestos exposure (>11.23 versus ≤11.23) by *iNOS *			
Crude	4.40	3.01–6.42	0.000
* iNOS* LL	3.09	1.81–5.25	0.000
Cumulative exposure: >11.23 versus ≤11.23			
* iNOS* SL + SS	5.74	3.30–9.99	
Cumulative exposure: >11.23 versus ≤11.23			

**Table 4:** Logistic regression analysis: interactions between *MnSOD* and *CAT*, *iNOS* and *CAT*,  *GSTM1* and smoking, *iNOS* and smoking, and *iNOS* and log-cumulative asbestos exposure.

	OR	95% CI	*P* value
*MnSOD* −9Ala/Val + Val/Val versus Ala/Ala	0.59	0.39–0.91	0.016
*CAT* −262 TT versus CT + CC	0.53	0.17–1.62	0.266
Interaction^†^	4.49	1.08–18.61	0.038
*iNOS* LL versus SL + SS	1.08	0.75–1.55	0.687
*CAT* −262 TT versus CT + CC	0.63	0.24–1.66	0.354
Interaction^‡^	4.78	1.15–19.81	0.031
*GSTM1*-null versus present	0.63	0.39–1.02	0.062
Smoking	0.55	0.32–0.96	0.036
Interaction^#^	2.67	1.31–5.46	0.007
*iNOS* LL versus SL + SS	0.85	0.53–1.37	0.505
Smoking	0.70	0.43–1.13	0.143
Interaction^§^	2.00	0.99–4.03	0.054
*iNOS* LL versus SL + SS	1.91	1.07–3.42	0.030
Log cumulative exposure	4.25	2.79–6.46	0.000
Interaction*	0.55	0.31–0.97	0.037

^†^Interaction: *MnSOD* −9Ala/Val + Val/Val versus Ala/Ala *CAT −262 TT versus CT + CC.

^‡^Interaction: *iNOS* LL versus SL + SS *CAT −262 TT versus CT + CC.

^#^Interaction: *GSTM1*-null versus present *smoking (ever/never). ^§^Interaction: *iNOS* LL versus SL + SS *smoking (ever/never).

*Interaction: *iNOS* LL versus SL + SS *log cumulative exposure.
